# What factors influence the retention of workers in NHS mental health crisis services in England? A reflexive thematic analysis

**DOI:** 10.1136/bmjopen-2025-104551

**Published:** 2025-10-13

**Authors:** Constance Hobbs, Emily Wood

**Affiliations:** 1 Health Education England Yorkshire and the Humber School of Public Health, Leeds, UK; 2 School of Medicine and Population Health, The University of Sheffield, Sheffield, UK

**Keywords:** MENTAL HEALTH, Health Workforce, QUALITATIVE RESEARCH, Health Services, Adult psychiatry

## Abstract

**Abstract:**

**Objectives:**

To understand factors that influence the intention of workers to remain in or leave employment in National Health Service (NHS) mental health crisis services and to use findings to formulate recommendations for NHS trusts to achieve improved worker stability in mental health crisis services.

**Design:**

A reflexive thematic analysis was conducted to explore the retention-related experiences of crisis workers. Secondary data was obtained from interviews conducted with crisis workers. This was collected by The University of Sheffield as part of the Retention of Mental Health Staff (RoMHS) study.

**Setting:**

Six NHS Trusts in England.

**Participants:**

All crisis worker interviews from the RoMHS study were included, totalling 10 participants: 70% female, 30% male, exclusively White British, and mostly occupying leadership roles.

**Results:**

Five themes were identified as influencing the retention of crisis workers: resource limitations, organisational culture and leadership, fairness and consistency, personal agency and team working. These themes are comparable to factors known to affect retention of the mental health workforce more widely. However, this study found a greater emphasis on the emotional burden of crisis work, including the challenges of complex risk management, and a perceived vulnerability of crisis service workers to increased workload and fewer development opportunities compared with other specialist mental health services.

**Conclusion:**

This study identifies that crisis workers face similar retention-related issues compared with the mental health workforce more widely, but with additional challenges related to the emotional intensity of the work and susceptibility of crisis services to resource constraints compared with other specialist mental health services. Further research should focus on employees who left crisis services, under-represented groups within the crisis workforce and the impact of retention-related policy.

**STRENGTHS AND LIMITATIONS OF THIS STUDY**

- Use of qualitative methodology and thematic analysis techniques allowed for an in-depth exploration of insights of participants into retention-related issues.
- Use of a voluntary sampling strategy presents a risk of selection bias, for example, by over-representing service managers compared with frontline staff.
- The sampling frame excluded crisis workers who had left employment, despite their relevance to the issue of retention.
- Only registered clinical staff working in National Health Service trusts in England were included, limiting wider applicability.
- The analysis, although conducted by a single researcher, incorporated reflexivity to address subjectivity and involved a second researcher for reviewing ambiguous codes to ensure interpretative rigour.

## Introduction

 The National Health Service (NHS) in England is facing a workforce crisis,^1^ characterised by high vacancy rates,^2^ an inadequate skill mix and projected increases in healthcare demand that are unlikely to be met by corresponding workforce growth.^3^ Staffing shortages negatively affect the quality, capacity and efficiency of healthcare service provision,^3^ as well as staff well-being.^4^

Mental health services consistently have higher vacancy rates than other NHS sectors, with one in 10 posts unfilled.^2^ They also face a greater projected rise in demand due to increasing rates of mental illness and health-seeking behaviour.^5^ The COVID-19 pandemic further exacerbated pressure on mental health services with predicted long-standing effects.^6^ The last dedicated national mental health workforce plan was published in 2017, with little evidence of its objectives being achieved.^7^ This may reflect wider inequalities in resource allocation and prioritisation between mental and physical health.^8^

Staff retention is critical to addressing current and projected workforce shortages, as noted in the NHS Long Term Workforce Plan.^3^ Poor retention is also linked to reduced quality of patient care, adverse outcomes and higher healthcare costs.^9^,^11^ Mental health services have among the lowest staff stability of all NHS trust types,^12^ with 12% of mental health workers having left in 2021/2022.^13^

Organisational culture, encompassing the overarching values, attitudes and behaviours of an organisation and its workforce,^14^ has been argued as key to retention of mental health workers.^15^ Retention is positively associated with well-managed organisational change, collaborative decision-making and fair pay and conditions.^15^,^19^ The provision of training and development opportunities demonstrates organisational investment in the mental health workforce and can improve retention by increasing the sense of value and confidence of workers.^15 16^ Other factors positively associated with retention of the mental health workforce include effective team functioning and relationships,^1620^,^23^ supportive line managers,^16^ and professional autonomy,^16 22 24^ which involves enabling workers to maintain self-governance^22^ and practise creatively.^25^

Poor staffing and high workload have been found to negatively impact the retention of mental health workers by reducing job satisfaction and increasing stress^4^ and moral distress.^15 16 26 27^ Moral distress describes the negative psychological effects that occur when professionals are restricted in their ability to take ethically correct actions due to external constraints.^27^ Emotional distress is a significant factor associated with increased intention to leave in mental health workers.^28 29^ This can occur from experiences of patient suicide, self-harm^20 30^ and aggression or violence.^16 20 21^ Proactive emotional regulation, such as self-care and maintaining professional boundaries, has been identified as a protective factor against these challenges among nurses in Norwegian and Australian mental health settings.^30 31^

It is acknowledged that staff in different mental health services may face distinct retention-related challenges, warranting further investigation on a setting-by-setting basis to develop targeted retention strategies.^16^ NHS Crisis Resolution and Home Treatment teams, hereafter referred to as ‘crisis teams’, provide around-the-clock, community-based care for individuals experiencing acute mental health needs, with an expected response time of a few hours. They visit individuals in their own homes, act as gatekeepers for inpatient beds and provide home treatment for those experiencing an acute psychiatric episode that would otherwise require hospital admission.^32^

The nature of this work could pose unique retention-related challenges compared with other areas of mental health, such as complex risk management^11^ and higher levels of emotional exhaustion.^23 33^ Despite this, no research specifically on the retention of the crisis mental health workforce in England was identified. Through analysis of interviews with crisis workers, this study aims to offer insights into the perspectives, experiences and challenges faced by this group concerning retention. The findings can be used to help shape targeted retention strategies for this setting.

## Methods

This paper describes a sub-analysis undertaken as part of a wider Retention of Mental Health Staff (RoMHS) study which has been reported elsewhere.^34 35^ The RoMHS study included case studies of six NHS trusts in England, consisting of staff interviews and a review of trust policies and procedures.

NHS trusts were identified purposefully with the aim of achieving sufficient variation across workforce stability, staff satisfaction, geographical region and urban-to-rural mix.^36^

Exclusion criteria included staff working in services for children and young people, non-clinical staff and agency staff. The team endeavoured to interview 4–6 senior staff and 25–35 frontline clinical staff at each site, with a minimum of 15 nurses and a maximum of 10 other professions, such as doctors, psychologists and social workers. Subsequent targeted emails were sent to under-represented groups, varying by Trust, such as staff from non-White backgrounds or specific professions.^36^

Interviews took place in 2021. They were conducted by EW and colleagues. Interviews took place on Microsoft Teams, Google Meet or by telephone, depending on the preference of the interviewee. Interviews were recorded on an encrypted digital recorder and transcribed using intelligent verbatim.

The interviews explored factors contributing to staff retention in mental health trusts. They were up to 1 hour long and carried out remotely due to COVID-19 restrictions. The interviews were semi-structured, with prompts based on key areas identified from stages I and II; workload and staffing, staff views on the care quality they can provide, leadership, teamwork, development opportunities, supervision and local context. Participants were asked about the impact of COVID-19 and compared with other trusts or sectors where relevant.^36^

The RoMHS study included 199 interviews across a variety of mental health teams, providing a useful overview of the retention-related issues relevant to mental health workers overall. However, it is suggested that different teams within mental health experience specific retention-related issues.^16^ These nuances may be lost in the overall analysis. This study therefore looked specifically at data from crisis teams.

Purposive sampling was used to identify relevant participants. The single inclusion criterion was staff working in crisis service settings. All grade and staff types were included, and no exclusion criteria were specified.

### Patient and public involvement

The RoMHS study consulted with two pre-existing mental health Patient and Public Involvement and Engagement groups in its design and set up. Participants were enthusiastic about the study as they felt that consistency of their worker was critical to their recovery.

## Analysis

Reflexive thematic analysis was conducted by CH, as per Braun and Clarke six-step guide:^37^ (i) familiarisation with dataset by reading and re-reading transcripts; this was of particular importance given the use of secondary data;^38^ (ii) generation of initial codes; (iii) generation of initial themes; initially, paper slips were used to provide a visual representation of codes, enabling creativity and exploration by rearranging slips to identify the most significant patterns; (iv) reviewing themes by codes and in relation to the entire dataset, using central organising concepts to provide clarity of the definition and boundaries of each theme; (v) defining and naming themes; and (vi) report write-up; a thematic mapping process was used to help visualise the relationships between themes and to provide an aid to report writing. Interview transcripts were uploaded onto NVivo (Lumivero, CO, USA) to help manage the data.

### Reflexivity and quality assurance

CH has personal experience transitioning from a frontline NHS role, meaning a reflexive approach was essential to understanding their relationship to the research. A reflexive journal helped name assumptions, verbalise experience of the analytical process, facilitate recognition of the subjectivity brought to the analysis and identify challenges as they arose, such as discussing ambiguous codes with EW.

To ensure academic rigour, elements of trustworthiness outlined by Lincoln and Guba^39^ were applied. Credibility was achieved by triangulating findings with existing literature and exploring notable differences. Transferability was supported through detailed descriptions of the study context, participants and methods. Confirmability was established by adopting a reflexive approach.

## Findings

Ten participants met the inclusion criterion (table 1). Almost half were from Trust F; however, each trust was represented by at least one participant. Trusts have been anonymised to protect participants. Participants were 70% female, which is similar to the NHS workforce, but exclusively of White British ethnicity, which does not reflect the ethnic diversity of the workforce.^40^ The sample included a range of roles, but with a higher proportion in leadership positions, particularly service management, compared with the overall NHS workforce structure.^41^ The average length of service of the participants in their current trust was 11 years.

**Table 1:** Participant characteristics

Pseudonym	Trust	Job title within crisis services	Ethnicity	Gender
A11	A	Service manager	White British	Male
A51	A	Consultant psychiatrist	White British	Female
B22	B	Clinical psychologist	White British	Female
C31	C	Clinical psychologist	White British	Male
D3	D	Psychiatric nurse	White British	Female
E34	E	Service manager	White British	Female
F12	F	Service manager	White British	Female
F13	F	Service manager	White British	Male
F25	F	Psychiatric nurse	White British	Female
F34	F	Psychiatric nurse	White British	Female

### Thematic analysis

The analysis elicited a total of five themes: *resource limitations*, *organisational culture and leadership*, *fairness and consistency*, *personal agency* and *team working*. A summary of the key findings is outlined in table 2.

**Table 2:** Summary of themes and subthemes

Theme/subtheme	Description
Theme 1: Resource limitations	The impact of restricted physical and mental resources on retention of crisis workers
Work intensity	The challenging nature of crisis work, characterised by heavy workloads, staffing shortages and subsequent psychological strain
Staffing levels and mix	The importance of well-staffed, balanced, stable teams for retention of crisis workers
Theme 2: Organisational culture and leadership	The influence of organisational culture and leadership on retaining crisis workers
Influence of leadership	The impact of leadership on retention through shaping workplace culture and allowing staff to feel valued
Change management	Interpretation, experiences and retention-related implications of effectively and ineffectively managed change
Theme 3: Fairness and consistency	The need for equal opportunities and experiences both within and across workplaces to support crisis worker retention
Theme 4: Personal agency	The role of personal agency in enabling work-related decision-making, navigating factors that limit choice and overcoming barriers to personal effectiveness and growth to promote crisis worker retention
Job autonomy	The value of opportunities to self-govern, with control and discretion over one’s working style and tasks, to promote crisis worker retention
Personal development	The value of opportunities for personal growth and development in supporting crisis worker retention
Theme 5: Team working	The value of strong teamwork, effective communication, and positive relationships within and across teams and professional groups to enhance crisis worker retention
Positive working within and between teams	The importance of effective teamwork for crisis worker retention, as well as the detrimental impact of conflict within or between teams
The relationship between the team and their direct line manager	The central role of direct line managers in supporting crisis worker retention

#### Theme 1: resource limitations

This theme covered the influence of both psychological and physical resource limitations on retention. The two subthemes were *work intensity* and *staffing levels and mix*.

##### Subtheme 1: work intensity

This subtheme explored the demanding nature of crisis work and the associated psychological burden, intensified by staffing shortages and increased workloads.

Challenges such as high referral rates, large caseloads and management of complex, high-risk patients were raised by some participants. The associated emotional burden of crisis work was seen as detrimental to retention:

We work with the most acutely unwell, and the most risky, we have a lot of suicides, a lot of coroner’s courts, it kills you. (E34)

Participants identified space and time as necessary resources to protect against the emotional intensity of crisis work. This included time for personal reflection, formal supervision and opportunities for informal debriefs:

The importance of thinking to the team and the space to think is the other thing…but this is something that I have been asked to be…cautious about exulting the virtues of because it can be misinterpreted as you’re doing nothing with your time. (F25)

Participants felt that high workload had been exacerbated by the COVID-19 pandemic, administrative duties and staffing shortages. High workload in crisis teams was seen to impede provision of quality care, damage staff well-being and subsequently increase the intention to leave:

They basically were just going out, making sure people had medication, saying, are you sleeping, are you eating, have you been to the toilet? Just awful questions like that, and then coming back, and that was the extent of their interaction with somebody who was at their most unwell and was trying to avoid hospital. (E34)

##### Subtheme 2: staffing levels and mix

This subtheme explored how staffing levels and skill mix influenced staff retention.

Participants described a vicious cycle in which staffing shortages negatively impacted well-being, leading to increased sickness and retention issues:

If it doesn’t feel like a bearable place to work, people will leave and then those who are left end up holding more and more until it becomes unbearable. (A51)

Conversely, well-staffed teams were seen to support staff retention:

That team is probably the happiest team out of all my teams at the moment, because…they’re actually fully staffed…I’m starting to actually retain staff in that team. (E34)

Participants stressed that an appropriate balance of skills and experience is essential for the multidisciplinary crisis team to function effectively. The loss or absence of team members, particularly leaders, was considered detrimental to effective working and subsequent retention:

Just having somebody who’s leading from a clinical perspective…I don’t think that particularly happens. ’Cause…with the team here, they haven’t had a permanent psychiatrist for like, probably coming up to over a year. (C31)

### Theme 2: organisational culture and leadership

This theme explored the impact of organisational culture and leadership on worker retention. Two subthemes were generated: *influence of leadership* and *change management*.

#### Subtheme 1: influence of leadership

This subtheme explored how organisational leadership shapes workplace culture, the role of leadership credibility in building trust and confidence, and the significance of leadership presence.

Most participants felt that organisational culture directly influenced workforce retention. Whether those hired into leadership positions were people-oriented or process-oriented was considered to affect the entire workplace culture:

The kind of staff they recruit gives a very strong message as to what their values are…as a trust board. (F12)

Values, attitudes and operations at organisational and leadership levels affected workforce retention. Compassionate leadership was important for fostering a positive workplace culture, and its absence was cited as a reason some considered leaving their posts:

I’ve seen staff being bullied by senior management and just leave because it’s not worth…people’s lives have been made misery. (D3)

Uncommunicative and unseen leadership was felt to reduce leadership credibility and sense of value among workers, thereby damaging retention:

Nobody knows what’s happening and think that the board don’t care about them. (E34)

Leaders described challenges in maintaining presence and effectively disseminating information to staff, such as working in a large trust. Email and online communication tools helped mitigate against this; however, their effectiveness was limited due to the high clinical workload of crisis teams:

They’re so busy in the crisis teams they don’t sit inside watching the computers…it’s almost like you need to have mandatory downtime every day just to read the Intranet. (F12)

#### Subtheme 2: change management

This subtheme explored the impact of change, both positive and negative, and the importance of managing change effectively. It highlighted the need for willingness and capacity to identify emerging problems, along with the ability to address them in a creative, collaborative and authentic manner.

Change was seen to have a long-standing impact on retention. Sometimes, change was considered necessary and constructive, particularly when it addressed the concerns of workers, was collaborative with staff or patients, or facilitated better teamwork:

The thing that…is just going really well is getting the whole system to come together to make decisions about the kind of care that they want…service users are at the centre, the staff views are shaping this…the whole point is that that is moving people towards work that gives them a sense of job satisfaction. (B22)

More often, however, frustrations over constant or recurrent change were evident, creating a sense of instability within the workforce and increasing the intention to leave. Historical instances of poorly managed change resulted in distrust among the workforce and aversion to future change:

They talk about that redesign, as if that’s still the thing that’s going on, 8 years later… And now, there’s another big redesign of the community services happening this year […] it’s almost like being retraumatised by the whole thing. (C31)

Participants described a need for leaders and systems that can promptly identify and address issues by fostering a culture in which leaders are accessible and approachable, and staff have opportunities to be heard. For example, a lack of exit interviews to identify retention-related issues was highlighted. Participants felt that, in some cases, problems were actively covered up through lack of senior leadership transparency and disincentives to whistleblowing:

I know friends who have raised issues and have had their fingers burnt and have been made to feel terrible because of what they’ve done. (D3)

In other instances, problems were passively ignored, as organisations selectively acknowledged only the issues that aligned with their agenda:

She didn’t even get a response saying thank you for your letter. Even like brushing you off…They pick and choose what they respond to. (D3)

Participants discussed the need for problems to be tackled in an effective way. Creative problem-solving was advocated to address large, complex problems, including inequality and staff shortages. Lack of ability to operate outside of organisational policy was perceived negatively:

I think sometimes the NHS can attract certain people where they can’t make a decision outside of a policy. That worries me. (A11)

Participants also emphasised that problems should be addressed in collaboration with staff. Top-down decision-making was felt to negatively impact retention by resulting in unreasonable demands of staff that showed lack of consideration for their perspective:

Why would you make…nurses from a team that’s working very well, go and do something that they really don’t like?…it just seems bonkers to me that on the one hand there’s all sorts of trust emails about retention…But what about retaining the people you’ve got? (D3)

### Theme 3: fairness and consistency

This theme addressed the need for equality of opportunity and experience across and within workplaces, regardless of specialty, role, location or individual leadership.

A lack of consistency in leadership and supervision quality was apparent, where having a good manager was considered a matter of luck. This inconsistency had implications for equity of working experience:

There will always be a difference in teams and how well they work because some people are going to be better as team leaders than others. (A51)

Participants highlighted inequalities between professional groups. Nurses in crisis teams were seen to be more vulnerable to organisational changes compared with smaller groups like occupational therapists:

It’s always my sense in the trust that OTs [occupational therapists]…seem a little bit more better protected and better treated, because I think they've got perhaps a stronger…control over their qualification…So they always seem to be able to manage to, yeah, to keep themselves more cohesive really. (D3)

Varying referral thresholds between comparable services across different areas were described, along with differences in staffing levels or provision. This created a sense of inequity among crisis staff. Specialist services were seen to have better workloads, investment and opportunities compared with crisis services:

I’ve known quite a few people who’ve jumped ship to perinatal services…There’s a lot of NHS England control over perinatal…so I think people are hoping, well, if I go there, it might not be as much nonsense. (D3)

Inconsistencies in pay, relative to agency workers and inflation, were highlighted. Others remarked that pay was not reflective of the intensity and responsibility of crisis work:

Do I really want to do this? I can earn more money filling shelves in Sainsbury’s. (E34)

Additionally, concerns about salary differences across geographical boundaries were frequently raised:

If you go a couple of miles over the border, you’re gonna be getting an extra few grand a year so that’s a, quite a, a sticking point for frontline staff. (F13)

### Theme 4: personal agency

This theme explored the importance of having the freedom to make work-related decisions, the factors that influence or limit choice, and the enablers and barriers to personal effectiveness and development at work. It included two subthemes: *job autonomy* and *personal development*.

#### Subtheme 1: job autonomy

This subtheme explored the value of having the opportunity to self-govern, with control and discretion over one’s working style and tasks.

Several participants expressed the need for autonomy in how they approached their duties. In handling significant risk, crisis workers valued the freedom to work without being micromanaged, while emphasising that autonomy should be offered alongside adequate guidance and support:

I just think if you’re a qualified clinician you can take that risk or you can take that decision and then I’ll back you as a manager…just giving people the autonomy to actually do their job…I think that’s quite rewarding to people. (A11)

Maintaining a role that suits the individual was identified as important for staff retention, including alignment with personal working preferences. Participants expressed frustration with generalised roles that led to deskilling:

I think going on an overly generic job descriptions and roles… the danger is that people then…become potentially professionally de-skilled. (F13)

Others felt there were limited opportunities to use their training and skills, often due to excessive administrative tasks:

I didn’t qualify to understand a flipping phone bill, and who’s paying for the bloody phone bill. I don’t care who’s paying for the phone bill. (E34)

Participants described how the size of trusts made changing employers impractical, which can lead to resentment:

There’s not the opportunity to move, if you’re in a massive patch like that. And you come across a lot of disgruntled people. (D3)

#### Subtheme 2: personal development

This subtheme considered the value of opportunities for personal growth and development in supporting worker retention.

Participants described the importance of training in helping staff feel valued and supported:

I think it can be quite…enticing to…stay within the Trust as you’ve got career development and progression opportunities. (F13)

Several participants also expressed concern about the lack of development opportunities for leaders. It was observed that it is often (wrongly) assumed that clinicians are naturally prepared for leadership roles simply by advancing in their clinical careers:

I think there’s something about…the training of managers, you know, it’s a really different, this is not rocket science, being a clinician and being a manager they are two completely different roles really and I think, as a whole, across the NHS, there’s insufficient recognition for that. (F25)

Participants also raised this issue in relation to supervision. They highlighted that while quality supervision is important for retention, there is limited training on how to deliver it effectively. This sometimes resulted in supervision of such poor quality that it was perceived as not worthwhile:

Really poor supervision is literally like a tick box exercise where they’re just speaking to you half an hour just chatting about your case load and it feels very corporate…I always think how can you expect someone to open up to you if they don’t know anything about you. (A11)

Several participants valued informal debrief opportunities more highly than supervision in receiving timely, practical and emotional support, and others sought support externally:

I end up seeing my own like therapist for supervision sometimes for the more emotional quality of the work…which feels a bit wrong. (F34)

### Theme 5: team working

This theme explored the importance of positive teamwork, communication and relationships both within and between different teams and professional groups. It included two subthemes: *positive working in and between teams* and *the relationship between the team and their direct line manager*.

#### Subtheme 1: positive working within and between teams

This subtheme highlighted the importance of effective teamwork for staff retention, as well as the detrimental impact of conflict within or between teams.

For crisis teams, effective teamwork, characterised by strong interpersonal relationships, mutual support and clear communication, was essential for managing complex risks, emotional intensity and rapid responses:

The work is incredibly stressful, so I think if you haven’t got…the team’s really important. (C31)

Participants discussed the need to recognise and address incompatibilities in working styles, ensure a fair distribution of workload and maintain clarity around individual responsibilities:

When you’ve just asked someone to do their job, they, my colleagues will be kind of like well why me…you’ve given me more work than everyone else, so yeah, so it’s a funny, an odd culture there. (F34)

Clashes between different professional groups were described by participants. For example, doctors were sometimes perceived as not practising holistically, and temporary staff were criticised for being driven by financial motives:

There’re too many problems with agency staff; there’s one that they’re not here for the right reasons. (A11)

As crisis teams work at the interface between inpatient and community settings, conflicts between teams were apparent. These often stemmed from system-level issues such as overwhelmed services:

We’ve had issues with the 4-hour response team not getting on with the team of staff in A&E [accident and emergency] because they do 1-hour response. (F12)

#### Subtheme 2: the relationship between the team and their direct line manager

This subtheme considered the central role of direct line managers in supporting staff retention.

Several participants emphasised the particular importance of this relationship. It was commonly observed that line managers often have greater impact on frontline workers than senior leadership:

I think the trust is fairly shocking in how it treats its staff…but I have been quite fortunate that I've obviously had very good line managers, which has allowed me to stay in those teams. (D3)

From the perspective of line managers, challenges in gaining team support were discussed. Some felt that being perceived negatively by their team was an inevitable part of a management role:

I hope that I’m not one of the leaders that they think, God, she’s rubbish, but they probably do, because that’s what happens, and I’ve kind of got to accept that. (E34)

## Discussion

High workloads combined with staffing inadequacies heightened stress among crisis workers and impaired their ability to provide quality care. This led to experiences of moral distress and an increased intention to leave, findings that are supported by wider research of mental health workers.^15^,^17^ The emotional burden of crisis work further added to these psychological demands, including witnessing distressing events and complex risk management. Similar challenges are identified in previous studies, such as fear of patient aggression^16^ and the distress of witnessing suicidal behaviour.^20 21 30^ In crisis settings, the burden of complex clinical decision-making and risk management is also highlighted.^11 33^

Space and time were essential psychological resources for some participants to mitigate against emotional exhaustion. This is more strongly supported by research situated in crisis settings,^17 33^ compared with other mental health contexts, where there is greater focus on the benefits of emotional regulation techniques.^30 31^ This could be explained by the specific psychological demands of crisis work, or, alternatively, by variability in the acceptability and prioritisation of this resource dependent on individual workplace culture. For instance, one participant was advised against publicising the benefits of reflective time for their team to avoid being perceived as unproductive.

Organisational culture and leadership was a large and pervasive theme in this study. Long *et al* found organisational culture to be the central determinant of mental health worker retention, from which other more specific issues stemmed.^15^ A reciprocal relationship was identified, supported by organisational theory,^14^ whereby leadership style and quality directly influenced workplace culture, which, in turn, enabled or impeded effective leadership. This study found that leaders struggled to maintain visibility across trusts, a challenge not prominent in the existing literature. This may be explained by the high proportion of leaders in the sample, or the heavy workload of crisis teams, which limits the time available for engaging with communications from leadership.

The working conditions in crisis services were compared unfavourably to those in other specialist services, like perinatal mental health, in terms of greater vulnerability to increased workload, funding differences and unequal training opportunities. Although the literature suggests that workers in community settings may face higher workloads and emotional demands,^23^ these differences are not well emphasised in existing research, due to the limited retention-related studies situated in crisis settings. Variability in experiences between and within workplaces, such as pay differences across trust boundaries, contributed to a sense of inequity among workers and increased motivation to leave in search of more favourable conditions elsewhere. This has been found in previous research, where it is emphasised that fair pay, rather than the salary itself, is important for mental health worker retention.^19^

Personal agency was seen to support retention, particularly through professional autonomy and development opportunities. While this is supported by research on mental health workers more widely,^16 22 24 42^ some evidence suggests that crisis teams offer greater autonomy compared with other mental health settings, contributing to higher levels of personal accomplishment and retention.^23^ Flexibility to change roles was sometimes restricted due to the excessive travel requirements imposed by the expanding size of trusts. Inability to leave a trust could lead to a harmful version of retention which fosters resentment, negatively impacting on team morale and quality of care. This appeared to be an evolving issue, which is not yet widely reported in the literature. This concerning development could be missed by statistical monitoring of workforce retention.

Effective teamwork was generally considered to encourage retention of crisis workers, aligning with findings from broader research on mental health staff.^16 22 43^ Participants also emphasised the need for cohesion between teams. This may be particularly significant for crisis workers given the challenges of working at the interface between community and inpatient settings.^11^ The role of the team’s direct line manager, and their relationship with team members, was identified as especially important for both effective team functioning and individual staff retention, and is supported in previous research.^16 25^ Concerns were raised about the inadequate training of leaders, particularly those with clinical or non-managerial backgrounds, which sometimes led to poor quality leadership and supervision. This issue is well recognised in existing literature.^44^,^46^

### Strengths and limitations

This study addressed an important and timely issue, with implications for future retention-related policies and practices. Thematic analysis permitted an in-depth exploration of the experiences and perspectives of workers. Although the study was conducted by a sole researcher, ambiguous codes were discussed with a second researcher, and a reflexive approach was used to account for subjectivity.

There may have been differences in levels of organisational engagement between volunteer participants and the wider workforce. Workers who had left their positions were not included, despite being a highly relevant group for understanding factors that affect retention.

There was no representation of ethnic minority groups or newly qualified workers, despite targeted efforts, which does not reflect the diversity of the NHS workforce^40 47^ and limits transferability.^48^ Over-representation of service managers may have skewed the study’s findings toward managerial experiences, limiting applicability to the wider NHS workforce. Other demographic data was not collected, such as participant age and disability status; therefore, it was not possible to determine how representative the sample was overall.

### Implications for policy and practice

These recommendations are aimed at NHS mental health trusts to improve retention of their crisis workforce:

1. Ensure crisis workers receive adequate emotional support and high-quality supervision to help manage the psychological burden of working with complex and high-risk patients.
2. Recognise that space and time are essential resources to mitigate against the high psychological burden of crisis work and improve well-being.
3. Promote visibility and effective communication between leadership and the wider crisis workforce through targeted strategies, such as flexible drop-in sessions, that accommodate the busy and irregular shifts of crisis workers.
4. Promote consistency in the working experience for crisis workers compared with other specialist mental health staff, ensuring fair distribution of resources and access to development opportunities.
5. Be mindful that the growing size of trusts may limit the ability of some crisis workers to change roles. Consider offering greater work-related choices within trusts, such as preferred patient groups or working schedules, to help mitigate this impact.
6. Invest in leadership training for crisis workers, particularly those from clinical or other non-managerial backgrounds, to foster supportive team relationships, ensure high-quality supervision and provide consistency across workplaces and teams.

### Future research

While this study offers some insights, given the paucity of retention-focused research in crisis settings, more research is needed to better understand the challenges faced by this group. Further studies should focus on groups that were under-represented in this study, including ethnic minorities, workers in non-leadership positions and those who are new to their trust, as well as those who have left crisis service employment. Research should also examine the long-term impact of the retention aspects of the NHS Workforce Plan and any subsequent changes to policy or practice, to help guide future strategies to promote retention of the NHS crisis workforce.

## Conclusion

Despite the shortage of published retention-focused research specific to crisis services, the findings of this study are comparable to studies which consider retention of the mental health workforce more widely. For example, retention was found to be impacted by organisational culture and leadership, opportunities for autonomy and growth, and effective teamwork. Adequate human and psychological resources were needed to mitigate against the risk of moral distress, as well as the emotional burden of crisis work which was emphasised in this study. In contrast to existing literature, this study found a perceived vulnerability of crisis workers to higher workload and inequitable development opportunities compared with other specialist mental health services, as well as challenges related to working in large trusts, including communication barriers and restricted freedom to move trust. Future retention-related research should focus on employees who left crisis services and specific groups within the crisis workforce, including ethnic minorities and newly qualified staff, to ensure that subsequent strategies are relevant and equitable across the workforce.

## Data Availability

No data are available.
