# Supplementary material for: What factors influence the retention of workers in NHS mental health crisis services in England? A reflexive thematic analysis
Source: BMJ Open. 2025 Oct 13;15(10):e104551. doi: 10.1136/bmjopen-2025-104551 (PMC12519645; doi:10.1136/bmjopen-2025-104551)
Supplement: Supplementary file 1 [file bmjopen-2025-104551.reviewer_comments.pdf]

PEER REVIEW HISTORY

BMJ Open publishes all reviews undertaken for accepted manuscripts. Reviewers are asked to complete a checklist review form and are provided with free text boxes to elaborate on their assessment. These free text comments are reproduced below.

ARTICLE DETAILS

Title (Provisional)

What factors influence the retention of workers in NHS mental health crisis services in England? A reflexive thematic analysis.

Authors

Hobbs, Constance; Wood, Emily

VERSION 1 - REVIEW

**Reviewer**	**1**
**Name**	**Scanlan, Justin**
**Affiliation**	**The University of Sydney, Faculty of Health Sciences**
**Date**	**07-Jul-2025**
**COI**	**None**

Thank you for the opportunity to review this manuscript. Overall, I believe that this is a useful study that provides a new focus on this important area. The unique experiences of mental health workers in crisis services has not received much attention i the literature to this point, so it is helpful to see this explored. The qualitative approach provides a nuanced understanding of the specific factors that may influence retention.

Overall, some of the themes presented feel "underdone" in terms of the detail provided in the representative quotes. While I recognise the limitations in terms of length of the paper, there are some places where it would be helpful to add additional quotes rather than including long lists of ideas and then providing only one quote. This was an issue over many of the descriptions of themes, but was most prominent for me in the Job Autonomy Section, for example "Maintaining a role that suits the individual was identified as important for staff retention, including alignment with personal working preferences. Participants expressed frustration with generalised roles that led to deskilling, limited opportunities to utilise their training and skills, excessive administrative tasks interfering with clinical duties, and trusts so geographically large that changing employers was impractical:" - there are many different ideas presented here, but then only one quote presented to support this range of ideas: "There's not the opportunity to move, if you're in a massive patch like that. And you come across a lot of disgruntled people (D3)"

Other points to be considered are in relation to the clarity of quotes presented:

Page 7, lines 41 to 44: "If the people at the top of the organisation are really thoughtful about the containment of staff that will feed down because they will expect that of the people the next layer down and they will expect that the next layer down. (A51)" - I am confused by this quote, particularly what is meant by "containment of staff".

Bottom of page 8: "If you're sending playing the game emails, like oh, tell me about this. But if you're actually addressing something like that, it…She didn't even get a response saying thank you for your letter…They pick and choose what they respond to. (D3)" -

It seems like "playing the game" might be some kind of workplace initiative, but it is not clear to me and I think needs some clarification to assist with understanding the context of the quote.

Bottom of p10: "there's less sort of proactive this is something that you need to have training in. (F25)" - I also find this a little confusing. Is the participant quoting someone (i.e., "there's less sort of proactive 'this is something that you need to have training in'. (F25)") or is there a pause in there? "there's less sort of proactive... this is something that you need to have training in. (F25)"

Page 13, line 31: "Effective teamwork was found to encourage retention of crisis workers" - this needs to be revised to be more speculative, as definitive statements like this that imply causation cannot be made from this dataset. Maybe something like "Effective teamwork was generally considered to encourage retention of crisis workers..."

A final consideration is the over-representation of service managers (4 out of 10 participants had this position title; and the authors reported that 70% were in senior leadership roles). As service managers are likely to have different perceptions of retention issues as compared with frontline staff, I think that this needs to be described in the limitations section along with some reflections on how this might have influenced the overall results of the study.

**Reviewer**	**2**
**Name**	**Leslie, Kathleen**
**Affiliation**	**Athabasca University, Faculty of Health Disciplines**
**Date**	**13-Aug-2025**
**COI**	**None**

Thank you for the opportunity to review this manuscript on retention of crisis workers in the NHS. This manuscript is part of a broader study on retention of mental health workers in the NHS and includes a subset of the qualitative interview data focused on crisis workers only. The article is clearly written and easy to follow.

Methods:

I suggest the authors include some additional details on methods for the interviews, including the following details:

- When were interviews conducted? Who conducted the interviews, and how many interviewees attended? What remote software was used? Were interviews recorded and transcribed?

- Who was included in the 'under-represented groups' that received targeted follow-up requests for interviews?

- Could authors clarify how many interviews were in the full study (the manuscript notes how many were sought for recruitment but not final numbers of included participants) so it is clearer what percentage of the full set of interviews these 10 participants represented?

- Could authors provide more details on the 'paper slips' used in analysis? Why was this manual process used in addition to NVivo software?

- Could primary author be more specific in their reflexivity? I am not sure what it means to have experience in "retention-related decisions". Does this mean CH worked in clinical leadership?

- Rather than stating 'elements of trustworthiness as outlined by Lincoln and Guba were applied', please be specific with what elements were applied and how.

Findings:

I'd suggest the authors highlight crisis workers and crisis settings in the results where possible. For example, with the finding that participants did not think that pay reflected the intensity of the work, the authors could note here that crisis work is particularly intense. Similarly, where the vicious cycle is described, the authors could note that this is particularly acute in crisis work where more workers left than in the broader mental health workforce. Under job autonomy and team working, the authors could relate this finding to the specifics of how work is conducted in crisis settings. The authors do a great job of this in the discussion, but I think adding a few specifics for crisis work in the findings will both differentiate this from the broader study thematic findings and transition smoothly into the implications for crisis workers described in the discussion.

I'd suggest a brief 'key findings' table with the themes and subthemes, a brief description, and potentially a representative quote might be a good addition at the beginning of the thematic analysis section.

I was interested in the finding that the larger group (nurses) were more vulnerable to organizational change than the smaller groups and wonder if the authors have any idea why that would be the case. Intuitively, I would have expected the opposite.

Other:

A few other corrections and suggestions:

- Suggest rewording page 3, line 45 for clarity in the exclusion criteria, e.g. “Exclusion criteria included staff working in child…”

- Define PPIE on first instance

- Could authors provide a brief description of crisis teams (what these teams do, how/where they respond to crises, where they work/types of crisis settings)?

- The subtheme ‘influence’ could be more clearly labelled perhaps as ‘influence of clinical leaders’ or similar

I hope these suggestions are helpful for the authors. Again, thank you for the opportunity to review this interesting paper.

VERSION 1 - AUTHOR RESPONSE

Reviewer 1

(1) Overall, some of the themes presented feel "underdone" in terms of the detail provided in the representative quotes. While I recognise the limitations in terms of length of the paper, there are some places where it would be helpful to add additional quotes rather than including long lists of ideas and then providing only one quote. This was an issue over many of the descriptions of themes, but was most prominent for me in the Job Autonomy Section, for example "Maintaining a role that suits the individual was identified as important for staff retention, including alignment with personal working preferences. Participants expressed frustration with generalised roles that led to deskilling, limited opportunities to utilise their training and skills, excessive administrative tasks interfering with clinical duties, and trusts so geographically large that changing employers was impractical:" - there are many different ideas presented here, but then only one quote presented to support this range of ideas: "There's not the opportunity to move, if you're in a massive patch like that. And you come across a lot of disgruntled people (D3)"
This suggestion has been addressed by including additional quotes in the findings section to better illustrate complex or multifaceted idees, particularly when a point encompasses several elements.

(2) Page 7, lines 41 to 44: "If the people at the top of the organisation are really thoughtful about the containment of staff that will feed down because they will expect that of the people the next layer down and they will expect that the next layer down. (A51)" - I am confused by this quote, particularly what is meant by "containment of staff".

This quote has been replaced with one that provides greater clarity, as reflected on page 8: “*The kind of staff they recruit gives a very strong message as to what their values are…as a trust board” (F12)*

(3) Bottom of page 8: "If you're sending playing the game emails, like oh, tell me about this. But if you're actually addressing something like that, it…She didn't even get a response saying thank you for your letter…They pick and choose what they respond to. (D3)" -
It seems like "playing the game" might be some kind of workplace initiative, but it is not clear to me and I think needs some clarification to assist with understanding the context of the quote.

A different excerpt from this quote has now been used, as the phrase 'playing the game' is unclear and not central to the point. The quote now reads "*She didn't even get a response saying thank you for your letter. Even like brushing you off...They pick and choose what they respond to"* as seen on page 9.

(4) Bottom of p10: "there's less sort of proactive this is something that you need to have training in. (F25)" - I also find this a little confusing. Is the participant quoting someone (i.e., "there's less sort of proactive 'this is something that you need to have training in'. (F25)") or is there a pause in there? "there's less sort of proactive... this is something that you need to have training in. (F25)"

It is not fully clear from the transcript which meaning the participant intended, so a different excerpt from this quote has been used; *I think there's something about…the training of managers, you know, it's a really different, this is not rocket science, being a clinician and being a manager they are two completely different roles really and I think as a whole across the NHS there's insufficient recognition for that"*, as seen on page 12.

(5) Page 13, line 31: "Effective teamwork was found to encourage retention of crisis workers" - this needs to be revised to be more speculative, as definitive statements like this that imply causation cannot be made from this dataset. Maybe something like "Effective teamwork was generally considered to encourage retention of crisis workers..."

This has been amended as suggested (page 15).

(6) A final consideration is the over-representation of service managers (4 out of 10 participants had this position title; and the authors reported that 70% were in senior leadership roles). As service managers are likely to have different perceptions of retention issues as compared with frontline staff, I think that this needs to be described in the limitations section along with some reflections on how this might have influenced the overall results of the study.

This has been made more explicit in the findings section on page 5 (regarding participant characteristics), as well as acknowledged in the limitations section on page 15.

Reviewer 2

Methods:

I suggest the authors include some additional details on methods for the interviews, including the following details:

(1) When were interviews conducted? Who conducted the interviews, and how many interviewees attended? What remote software was used? Were interviews recorded and transcribed?
Interviews took place in 2021. They were conducted by EW and colleagues. Some interviews took place on MS Teams or Google Meet, others over the telephone. This was the interviewee's preference. Interviews were recorded on an encrypted digital recorder and transcribed intelligent verbatim.

This has been added to page 3/4 of the methods section.

(2) Who was included in the 'under-represented groups' that received targeted follow-up requests for interviews?
This varied in the different Trusts. In some we did not get much initial response from inpatient services. In some it was certain professions. In some it was staff from non-White backgrounds. In some Trusts it was more than one group.

This has been added to page 3 of the methods section.

(3) Could authors clarify how many interviews were in the full study (the manuscript notes how many were sought for recruitment but not final numbers of included participants) so it is clearer what percentage of the full set of interviews these 10 participants represented?
199 interviews were conducted in total across all areas of mental health. The 10 included in this report represent all participants working in crisis and home treatment services.

This has been added to page 4 of the methods section.

(4) Could authors provide more details on the 'paper slips' used in analysis? Why was this manual process used in addition to NVivo software?
Paper slips were used to provide a visual representation of the codes, enabling creativity and exploration by rearranging them to identify the most distinctive and important patterns.

This further explanation has been added to the analysis section on page 4.

(5) Could primary author be more specific in their reflexivity? I am not sure what it means to have experience in "retention-related decisions". Does this mean CH worked in clinical leadership?

CH has not worked in clinical leadership but has moved from a frontline NHS role to a non-clinical position. This has been made more explicit in the 'reflexivity and quality assurance' section on page 4: "*CH has personal experience transitioning from a frontline NHS role, meaning a reflexive approach was essential to understanding their relationship to the research.*"

(6) Rather than stating 'elements of trustworthiness as outlined by Lincoln and Guba were applied', please be specific with what elements were applied and how.

Further detail on this point has been added to the 'reflexivity and quality assurance' section on page 5: "*Credibility was achieved by triangulating findings with existing literature and exploring notable differences. Transferability was supported through detailed descriptions of the study context, participants and methods. Confirmability was established by adopting a reflexive approach.*"

Findings:

(7) I'd suggest the authors highlight crisis workers and crisis settings in the results where possible. For example, with the finding that participants did not think that pay reflected the intensity of the work, the authors could note here that crisis work is particularly intense. Similarly, where the vicious cycle is described, the authors could note that this is particularly acute in crisis work where more workers left than in the broader mental health workforce. Under job autonomy and team working, the authors could relate this finding to the specifics of how work is conducted in crisis settings. The authors do a great job of this in the discussion, but I think adding a few specifics for crisis work in the findings will both differentiate this from the broader study thematic findings and transition smoothly into the implications for crisis workers described in the discussion.
More references specific to crisis workers have now been added to the findings section as suggested.

(8) I'd suggest a brief 'key findings' table with the themes and subthemes, a brief description, and potentially a representative quote might be a good addition at the beginning of the thematic analysis section.

A table summarising the themes and subthemes has been added at the beginning of the thematic analysis section (page 6). Representative quotes have not been included in the table, as it was considered more effective to present them within the text alongside the discussion. Additional quotes have also been incorporated into the findings section in response to Reviewer 1's feedback.

(9) I was interested in the finding that the larger group (nurses) were more vulnerable to organizational change than the smaller groups and wonder if the authors have any idea why that would be the case. Intuitively, I would have expected the opposite.

The participant quoted went on to speculate that this was because the smaller professional groups are able to maintain greater group cohesion and greater control over their qualification: *"It's always my sense in the trust that OTs…always seem a little bit more better protected and better treated, because I think they've got perhaps a stronger…control over their qualification…So they always seem to be able to manage to, yeah, to keep themselves more cohesive really".* This quote has now been expanded to include this suggested explanation (page 10).

Other:
A few other corrections and suggestions:

(10) Suggest rewording page 3, line 45 for clarity in the exclusion criteria, e.g. "Exclusion criteria included staff working in child…"

This has been amended as suggested (page 3).

(11) Define PPIE on first instance
This has been amended as suggested (page 4)

(12) Could authors provide a brief description of crisis teams (what these teams do, how/where they respond to crises, where they work/types of crisis settings)?

Additional details have been added to the introductory paragraph on Crisis Resolution and Home Treatment Teams on page 3:

"NHS Crisis Resolution and Home Treatment teams, hereafter referred to as 'crisis teams' provide around-the-clock, community-based care for individuals experiencing acute mental health needs, with an expected response time of a few hours. They visit individuals in their own homes, act as gate-keepers for inpatient beds, and provide home treatment for those experiencing an acute psychiatric episode that would otherwise require hospital admission[32]."

(13) The subtheme 'influence' could be more clearly labelled perhaps as 'influence of clinical leaders' or similar

This has been amended to *"Influence of leadership"* (page 8).

VERSION 2 - REVIEW

**Reviewer**	**1**
**Name**	**Scanlan, Justin**
**Affiliation**	**The University of Sydney, Faculty of Health Sciences**
**Date**	**21-Sep-2025**
**COI**	

All of my concerns have been addressed. Thank you for your updates.

**Reviewer**	**2**
**Name**	**Leslie, Kathleen**
**Affiliation**	**Athabasca University, Faculty of Health Disciplines**
**Date**	**15-Sep-2025**
**COI**	

Thank you to the authors for the fulsome responses and careful revisions. My suggestions have been adequately addressed, and I believe the paper can be accepted for publication.
